# Similar EEG Activity Patterns During Experimentally-Induced Auditory Illusions and Veridical Perceptions

**DOI:** 10.3389/fnins.2021.602437

**Published:** 2021-04-01

**Authors:** Maryam Faramarzi, Florian H. Kasten, Gamze Altaş, André Aleman, Branislava Ćurčić-Blake, Christoph S. Herrmann

**Affiliations:** ^1^Experimental Psychology Lab, Department of Psychology, European Medical School, Cluster of Excellence “Hearing4All,” Carl von Ossietzky University, Oldenburg, Germany; ^2^Department of Biomedical Sciences of Cells and Systems, Cognitive Neuroscience Center, University of Groningen, University Medical Center Groningen, Groningen, Netherlands; ^3^Neuroimaging Unit, European Medical School, Carl von Ossietzky University, Oldenburg, Germany; ^4^Research Center Neurosensory Science, Carl von Ossietzky University, Oldenburg, Germany

**Keywords:** auditory perception, auditory illusions, auditory awareness negativity, CPP, late positivity, P300, SCP, signal detection

## Abstract

Hallucinations and illusions are two instances of perceptual experiences illustrating how perception might diverge from external sensory stimulations and be generated or altered based on internal brain states. The occurrence of these phenomena is not constrained to patient populations. Similar experiences can be elicited in healthy subjects by means of suitable experimental procedures. Studying the neural mechanisms underlying these experiences not only has the potential to expand our understanding of the brain’s perceptual machinery but also of how it might get impaired. In the current study, we employed an auditory signal detection task to induce auditory illusions by presenting speech snippets at near detection threshold intensity embedded in noise. We investigated the neural correlates of auditory false perceptions by examining the EEG activity preceding the responses in speech absent (false alarm, FA) trials and comparing them to speech present (hit) trials. The results of the comparison of event-related potentials (ERPs) in the activation period vs. baseline revealed the presence of an early negativity (EN) and a late positivity (LP) similar in both hits and FAs, which were absent in misses, correct rejections (CR) and control button presses (BPs). We postulate that the EN and the LP might represent the auditory awareness negativity (AAN) and centro-parietal positivity (CPP) or P300, respectively. The event-related spectral perturbations (ERSPs) exhibited a common power enhancement in low frequencies (<4 Hz) in hits and FAs. The low-frequency power enhancement has been frequently shown to be accompanied with P300 as well as separately being a marker of perceptual awareness, referred to as slow cortical potentials (SCP). Furthermore, the comparison of hits vs. FAs showed a significantly higher LP amplitude and low frequency power in hits compared to FAs. Generally, the observed patterns in the present results resembled some of the major neural correlates associated with perceptual awareness in previous studies. Our findings provide evidence that the neural correlates associated with conscious perception, can be elicited in similar ways in both presence and absence of externally presented sensory stimuli. The present findings did not reveal any pre-stimulus alpha and beta modulations distinguishing conscious vs. unconscious perceptions.

## Introduction

Classically, the brain was viewed as a processing unit which passively translates sensory inputs into perceptions. In this traditional account, sensory processing and perception were considered as a feature extraction and reconstruction algorithm implemented in hierarchical neural structures (Engel et al., 2001). However, further developments in the field have shown that sensory perception is not merely driven by stimulus properties but the brain acts as an active inference device which influences perception based on its existing internal state (Kersten et al., 2004). In other words, perception of sensory inputs is shaped by prior expectations and attentional and working memory states (Summerfield and Egner, 2009; Kok et al., 2013).

The balanced interaction of feedforward information transfer from external sensory inputs (bottom-up) and the feedback coming from brain’s predictions (top-down) results in what has been referred to as normal perception in previous studies (Powers et al., 2016). Perturbation in normal balance of the corresponding mechanisms has been hypothesized to give rise to perceptual abnormalities such as hallucinations, i.e., perceptions in the absence of a corresponding external stimulus (Friston, 2005) and illusions, i.e., perceptions that deviate from the original stimulus (Langguth et al., 2013). Studying the neural mechanisms underlying these experiences helps us elucidate both basic and pathological mechanisms underlying normal and abnormal perceptions.

Even though experiencing illusions and hallucinations is mostly associated with psychotic disorders and has been prominently studied in the psychiatric patient populations (Silbersweig et al., 1995; Sritharan et al., 2005; Kindler et al., 2011), it is not restricted to clinically diagnosed patients as evidenced by many reports from otherwise healthy populations (Sommer et al., 2010; Baumeister et al., 2017). Moreover, auditory illusions and hallucinations can be elicited in healthy subjects by means of suitable experimental paradigms (Bentall and Slade, 1985; Powers et al., 2017), potentially resulting from bottom-up manipulations, such as hyperactivation of the auditory cortex, and/or modulation of top-down processes, such as increased levels of prior expectation on hearing of an auditory stimulus (Waters et al., 2012; Hoskin et al., 2014; Powers et al., 2016).

Some authors propose that there is a continuum from normal to aberrant perception. This implies that electrophysiological networks underpinning hallucinations and illusions in clinical populations might as well underly similar perceptual experiences in healthy subjects (Barkus et al., 2007; van Os et al., 2009; Larøi, 2012; Larøi et al., 2012). On the other hand, studying such phenomena in non-clinical populations is favorable, due to the absence of confounds such as co-morbidities and effects of medication (Diederen et al., 2012; Waters et al., 2012). Therefore, investigating experimentally-induced hallucinations or illusions in healthy subjects can serve as a viable alternative for examining these phenomena in patient populations and opens up another avenue to explore their underlying neural processes. In the present study, we focus on the relationship of our results to previous research on the neural correlates associated with conscious auditory perception and experimentally-induced illusions and hallucinations and we do look into the neural studies on hallucinations and illusions as pathological conditions.

A number of experimental paradigms have been utilized to induce different forms of experimental illusions and hallucinations. For instance, an auditory signal detection task has been introduced in which ambiguous auditory stimuli at near detection threshold intensity embedded in noise were presented and subjects reported hearing speech snippets in their absence (Bentall and Slade, 1985). Auditory continuity illusion is another form of illusion which refers to the phenomenon of hearing an interrupted sound in noise as continuous (Riecke et al., 2011, 2012). Taking advantage of the Pavlovian learning paradigm, task-elicited auditory hallucinations can be generated by first pairing a visual with an auditory stimulus and then removing the auditory stimulus (Powers et al., 2017). The Zwicker tone illusion is another form of auditory illusion, described to occur after presenting a notch-filtered broad-band noise. The heard tone has a pitch equal to the suppressed frequency band of the preceding notch-filtered noise (Zwicker, 1964).

The aforementioned paradigms have been utilized in several studies to address various behavioral and neurophysiologic questions including investigations on the underlying cortical processes involved in task-elicited auditory illusions. Alpha oscillations have been shown to play an important role in the formation of illusions in different sensory modalities including auditory domain (for a review see Lange et al., 2014). Reduced alpha power has been found during experimentally-induced auditory illusions, most prominently over temporal regions in a speech in noise signal detection task (Schepers et al., 2016). A relative increase in upper alpha power with respect to baseline was observed during the Zwicker tone illusion along with a linearly decreasing alpha power trend with increasing levels of perceived loudness of the tone (Leske et al., 2014). The patterns observed in the alpha band in this study were generally extended to beta frequency ranges. On the other hand, low-frequency power and phase locking have been implicated in the experience of auditory continuity illusion. The auditory continuity illusion was accompanied by suppression of cortical power and the phase locking at ∼3–4 Hz which was hypothesized to account for the blurring of auditory object boundaries that results in continuous perception of the auditory input (Riecke et al., 2009, 2012; Kaiser et al., 2018). A similar pattern albeit stronger has been found in patients with schizophrenia (Wooldridge et al., 2020).

Previous studies suggest that two ERP components are involved in near-threshold stimulus awareness. Firstly, an early negativity called visual awareness negativity (VAN) in the visual domain (Koivisto and Revonsuo, 2010; Rutiku et al., 2015) and auditory awareness negativity (AAN), its counterpart in the auditory domain (Eklund and Wiens, 2019; Eklund et al., 2019, 2020) have been observed in aware trials in contrast to unaware trials. This component was followed by an LP, similar to P300 which has been proposed as one of the most prominent neural markers of conscious perception (Dehaene and Changeux, 2011; Rutiku et al., 2015). The classic P300 has been suggested to be equated to the proposed decision variable signal in humans, called centro-parietal positivity (CPP), which identifies the dynamics of the decision formation (O’Connell et al., 2012; Kelly and O’Connell, 2015; Twomey et al., 2015). In perceptual decision-making theories, the decision variable integrates noisy sensory evidence and determines decision through a boundary-crossing decision criterion (O’Connell et al., 2012). The classic P300 as the peak of a unitary event, can be viewed as the peak that is formed when this decision variable signal reaches a boundary threshold, and returns back to baseline (Kelly and O’Connell, 2013; Twomey et al., 2015). A remarkably close relationship has been observed between CPP and the subjective perceptual experience (Tagliabue et al., 2019) and a reliable build-up in CPP for false alarms (FA) was observed in a gradual visual target detection task (O’Connell et al., 2012).

In the present study, we employed an auditory signal detection task to induce auditory illusions in a sample of healthy subjects. In order to experimentally-induce illusions, we presented speech signals near detection threshold embedded in speech-shaped noise. The inter-stimulus interval (ISI) was varied in a controlled fashion to alter participants’ expectations to hear a speech snippet, thereby inducing illusory auditory perceptions, which were captured in the trials where no external stimulus was presented but a response was given. Our aim was to investigate the elicited ERP components as well as ERSPs in different frequency bands (delta to beta) in false alarms (FAs) by comparing the activity in the activation period vs. baseline period. We further compared activities across different conditions. FAs were compared to hits where an externally presented stimulus caused a response to assess the activity differences in illusory and veridical perceptions. Furthermore, FAs were compared to trials where no response was triggered in the absence of stimuli (correct rejection, CR) as well as where motor responses were given without any perceptual experience (button press, BP) to account for the effects caused by the action of pressing the button.

## Materials and Methods

### Participants

Twenty volunteers participated in the auditory signal detection experiment (10 females, age: 25.84 ± 3.01 years) and a subset of 10 performed an additional block of control button press task. All participants were students at the University of Oldenburg, had sufficient English language proficiency to understand the instructions, and received monetary compensation for their time. The participants were right-handed according to Edinburgh handedness inventory (Oldfield, 1971), had normal hearing and none of them reported any history of neurological or psychiatric disorders. Written informed consent was obtained from every participant prior to the experiment. The experimental protocol was approved by the “Commission for Research Impact Assessment and Ethics” at the University of Oldenburg. The study was called an auditory signal detection task and the participants were unaware of the purpose and background of the study during the experiment. Three participants having insufficient FAs (<10) after removing artifactual trials were excluded from EEG analysis. A further participant was excluded due to excessive muscle and movement related artifacts. In total, sixteen participants were included for the EEG analysis (7 females, age: 26.83 ± 2.67 years). Seven of these were among the participants who took part in the control button press task (3 females, age: 26.29 ± 2.32 years).

### Paradigm: Auditory Signal Detection Task

**FIGURE 1:**
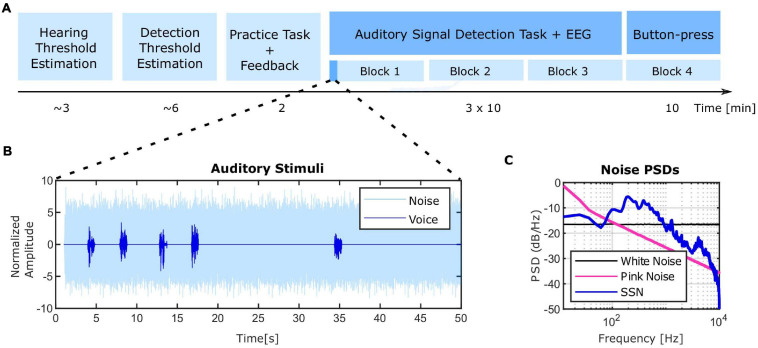
Experimental design and stimuli. **(A)** Timeline of the experimental session. At the beginning, each individual’s hearing threshold for the masking noise was estimated (∼3 min). The noise intensity level was set to 40 dB HL and the speech snippet intensity leading to a 70.7% correct detection rate was estimated in the next part (∼6 min). The speech intensity was set within ± 1.5 dB range of the detection threshold. A short practice task was implemented before the main experiment at which positive feedback was provided for correct detections and negative feedback for missed detections of the stimuli (2 min). Afterward, the main signal detection experiment started which was divided into three blocks with self-paced breaks in between while EEG was recorded. Finally, a subset of subjects took part in an additional task of presseing the button as a control condition (more detailed information can be found in “Materials and Methods” section). **(B)** The amplitude of speech and noise stimuli over a sample 50 s sub-period of the total block duration. The same ISI pattern was repeated twelve times in the course of each experimental block (10 min). **(C)** Power spectral density (PSD) of the speech-shaped-noise (SSN) used in the experiment in comparison to white and pink noise. SSN was used as the masking noise in the present study.

We employed the general framework of auditory signal detection task developed by Bentall and Slade (1985) and further used by others (Barkus et al., 2007; Moseley et al., 2014) to prompt experimentally-induced auditory illusions in healthy participants. The current experimental paradigm was carried out in four steps (Figure 1A), measurement of a rough individual hearing level, measurement of the speech in noise detection level, a training task and the signal detection task which was distributed over three blocks with self-paced breaks in between.

#### Auditory Stimuli

In the original experiment by Bentall and Slade (1985), a single word was used in the entire duration of the experiment. We used different speech stimuli similar to Barkus et al. (2007, 2011), Moseley et al. (2014) to increase the difficulty of the task. For the same reason, in contrast to an androgynous voice used in previous studies, voices from different male and female speakers were employed. The speech stimuli were comprised of 1 s snippets of English audiobooks, from natural voices of four female and four male speakers. The following steps were then followed to create the final speech stimuli set. Firstly, a subset of speech snippets was subjectively selected from a larger data set for not having long silent periods or high intensities. Afterward, the speech snippets were normalized to the root mean squared (rms) power. Finally, onsets and offsets were smoothed with a 100 ms Hann window. In total, 60 speech stimuli were used for the main experiment. In order to make the final auditory stimuli, speech stimuli were embedded in steady-state speech shaped noise (SSN) (Wilson et al., 2007). SSN was constructed by taking the Fourier transform of all the speech snippets, randomizing the phases and converting them back to the time-domain by doing an inverse Fourier transform. Higher masking effect can be achieved by SSN instead of more widely employed white (Barkus et al., 2007; Moseley et al., 2014) and pink noises (Moseley et al., 2016; Figure 1C) because of the higher overlap of the speech and noise spectra. Speech and noise signals were resampled to 50 kHz to be compatible with hardware requirements of our setup.

The final auditory signal was loaded in Matlab and streamed to a NIDAQ (National Instruments Data Acquisition, National Instruments, Austin, TX, United States) used for the digital-to-analog conversion. The analog signal was transmitted to a programmable Attenuator (PA5, Tucker Davis Technologies, TDT, Alachua, FL, United States) and then fed to a headphone driver (HB7, Tucker Davis Technologies, TDT, Alachua, FL, United States). E-A-RTONE Gold 3A Insert Earphones (3M Auditory Systems, Indianapolis, United States) were used to present the auditory stimuli. Auditory stimuli were presented binaurally throughout the experiment. Presentation of visual information on the screen and collection of the participants’ responses were achieved using Psychophysics Toolbox Version 3 (PTB-3, 2009) (Brainard, 1997) running on Matlab 2018a (The MathWorks Inc., Natick, MA, United States). We aimed to determine an individually titrated threshold for detecting the speech snippets in noise. To this end, we conducted two stages of hearing level measurements as described below.

#### Hearing Threshold Estimation

In order to set the intensity of the noise according to each individual’s hearing abilities, prior to the experiment, the individual hearing threshold for each subject was measured with a self-adjustable adaptation procedure (Baltus and Herrmann, 2015). We conducted the measurement with 400 ms bursts of noise (instead of pure tones) to determine a frequency unspecific estimate of the hearing level. In brief, the noise level started at a clearly audible level and decreased in steps of 2 dB until the subjects reported with a button press that they are not hearing the noise anymore. Then, we subtracted 10 dB from the mentioned level and presented the stimulus with gradually increasing sound pressure level in steps of 2 dB until the subjects were able to hear it again. This procedure was repeated three times and the mean value of the noise levels at the time of the subjects’ reports of “hearing again” and “not hearing anymore” were taken as individual hearing level (IHL). The estimated IHL was then used to adjust the masking noise level in the main experiment at 40 dB above IHL.

#### Speech Detection Threshold Estimation

Once the noise intensity was determined and fixed, the intensity of the speech stimuli was adjusted individually. This procedure was similar to the speech reception threshold (SRT) estimation that aims to identify the signal-to-noise ratio (SNR) that yields on average 50% correct recognition over a number of trials (Dingemanse and Goedegebure, 2019) with two differences. Firstly, we were not interested in recognition of speech snippets but only in their detection in noise and secondly, we aimed for 70.7% detection threshold instead of 50%. We selected our range of amplitudes around 70.7% threshold for two reasons. Firstly, it was desired that the stimuli became perceived with the ISI that they were presented as much as possible, since this pattern of ISI was intended to modulate the expectation to hear voices and therefore to increase FAs. For this purpose, selecting a higher threshold was advantageous. Secondly, selecting a higher threshold provided the participants more confidence in their responses and avoided the task getting too hard to induce very high guessing rates and random responses. A fast procedure was implemented to measure a rough estimate of the individual speech detection threshold using an adaptive staircase procedure with a simple 1 up / 2 down rule aiming at a 70.7% detection threshold (Shen, 2013). In this task, the noise intensity was set and presented continuously at 40 dB above IHL as measured in the previous step and speech snippets were randomly interspersed in noise with 3–6 s inter-stimulus interval (ISI). The participants were asked to respond by a button press whenever they detected a speech snippet. The step size was initialized at 1 dB and was decreased to 0.5 dB after four reversals and the whole procedure stopped after nine reversals. The average of the speech intensity at the last five reversal points was calculated as the speech intensity of 70.7% detection threshold. The intensity of the speech stimuli in the main experiment was then selected by drawing random samples from a uniform distribution in the ± 1.5 dB range of the speech intensity at the detection threshold.

#### Practice Task

Preceding the main auditory signal detection task, participants performed a short training task with online feedback, in order to get familiarized with the main task. Correct detection of speech was followed by a message “Correct” appearing on the screen and misses with a “You missed it.” message. No feedback (neither positive nor negative) was provided to FAs.

#### Main Experiment

The main experiment followed immediately afterward. The stimuli consisted of a 30 min stream of noise in which 180 speech stimuli were pseudo-randomly interspersed at 3–19 s ISI and presented in three blocks with a duration of 10 min. Each 10 min block was virtually composed of 12 sub-blocks of 50 s length with identical ISI settings which were shorter at the beginning and increased toward the end (Figure 1B). This manipulation aimed at increasing the expectation of participants to hear frequent speech snippets in the beginning of each segment which in turn caused them to experience more false perceptions in the next sub-period which in fact had less frequent speech stimuli. The participants were instructed to press the spacebar each time they detected a speech snippet.

#### Button-Press Task as a Control Condition for Motor-Related Processes

To control for movement-related activity during FAs, a subset of subjects performed an additional button-press (BP) task after the main experiment. The same noise as in the main experiment was presented during the 10 min duration of the task. Speech snippets were presented every 10 s with ± 1 s onset-jitter. The intensity of the speech stimuli was set at a higher level than the main experiment to make it clearly audible. More specifically, it was randomly drawn from a uniform distribution in the 5–6 dB range above the speech intensity at each individual’s detection threshold. The participants were asked to respond approximately 5–6 s after hearing the speech stimuli. This time interval between pressing the button and the speech stimuli was chosen to ensure sufficient separation between the corresponding neural activations related to hearing the speech stimuli and the button presses. The participants were asked to avoid a silent counting strategy for responding. A total of 60 stimuli was presented and 60 responses were collected.

### EEG Recording

The experiment was performed in a sound attenuated, electrically shielded room which was dimly illuminated with a battery-driven LED lamp. Participants were seated in a reclining chair in front of a computer screen and were asked to fixate on a cross in the center of the screen while performing the auditory signal detection task. EEG activity was recorded from 64 Ag-AgCl electrodes mounted in an elastic cap (EasyCap, GmbH, Herrsching, Germany) following the international 10-10 system layout. The reference electrode was connected to the nose and the ground electrode was positioned at AFz. An electrooculogram (EOG) was recorded from underneath the right eye. Electrodes were filled with an electrically conductive, abrasive gel called Abralyte (Brain Products GmbH, Gilching, Germany). Impedances were kept below 10 kΩ during the experiment. EEG was measured and recorded at a sampling frequency of 1,000 Hz via a BrainAmp amplifier (Brain Products GmbH, Gilching, Germany) and digitally stored on a computer using Brain Vision Recorder Software (Brain Products GmbH, Gilching, Germany).

### Analysis

Data analysis was performed using Matlab 2019a (The MathWorks Inc., Natick, MA, United States) and the Fieldtrip toolbox (Oostenveld et al., 2011).

#### Behavioral Analysis

**FIGURE 2:**
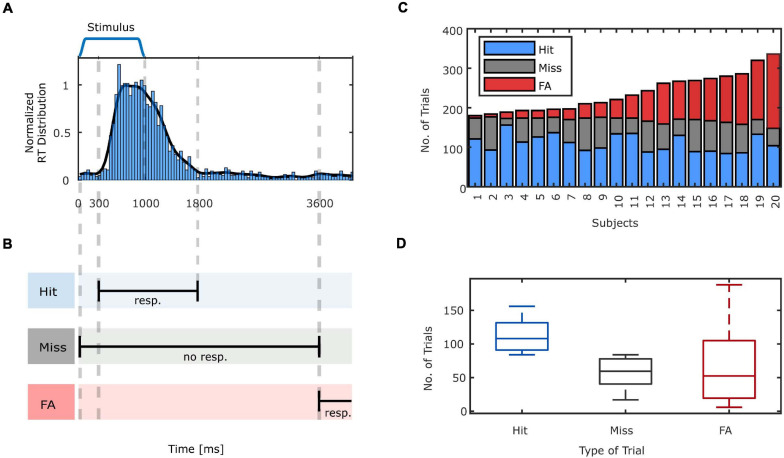
**(A)** The reaction time (RT) histogram, normalized to produce an estimation of the probability density function (PDF) and Gaussian smoothed distribution for all subjects relative to stimulus presentation. **(B)** Criteria for categorizing the trials. A hit is identified when a response is given to a stimulus in the time interval between 300 and 1,800 ms. If no response is given to the stimulus up to 3,600 ms, this trial is labeled as a miss. Responses that occur in a time window lasting from 3,600 ms after stimulus presentation until the onset of the next stimulus denote FA trials. **(C)** The number of hits, misses and FAs across the subjects. **(D)** The box plot of the number of hits, misses and FAs for all subjects. On each box, the central mark indicates the median, and the bottom and top edges of the box indicate the 25th and 75th percentiles, respectively. The whiskers extend to the most extreme data points not considered outliers.

As the experiment was designed in a continuous format, there were no predefined time intervals for the response collection. Hence, it was necessary to identify which responses were given to the preceding stimuli (hits) and which were not related to speech stimuli (FAs). On the one hand, the minimum response time to the auditory stimuli is bounded by the time needed for the physiological processes involved in stimulus perception and motor movement execution (Whelan, 2008). On the other hand, participants were instructed to respond promptly to the stimulus, thus, very late responses to stimuli were unlikely to be initiated by the preceding stimuli. In order to obtain the appropriate criteria to classify participants responses as hits and FAs, the histogram of the response times to speech stimuli across all subjects with a bin length of 42 ms were calculated (Figure 2A). Based on the smoothed histogram (Gaussian-weighted moving average filter, window length equal to 420 ms), hits were defined if a response was given in the time interval of 300–1,800 ms post stimulus onset. The same criterion translates to response-aligned hits as the responses that are preceded by a stimulus onset in the −1,800 to −300 ms time interval pre-response. The trial was marked as a miss if no response was given in the 3,600 ms interval post-stimulus onset. The margin of 1,800 ms was chosen in order to not categorize late response hits as misses. Additionally, in order to not mistakenly categorize a late response to a stimulus as a FA, we considered 1,800 ms marginal boundary between the latest valid response to a stimulus and a response that is presumably not initiated by the preceding stimulus. More specifically, a trial was marked as FA, if no stimulus onset occurred in the −3,600 ms pre-response interval (Figure 2B). Setting a marginal boundary for the criteria for categorizing trials, is aimed to increase the classification accuracy and hence a better segregation of the neural patterns in illusory from veridical as well as conscious from unconscious perceptions. The remaining parts of the EEG that were not classified as hits, misses or FAs were categorized as CRs.

#### EEG Analysis

##### Preprocessing

The EEG data was processed using the Fieldtrip toolbox (Oostenveld et al., 2011). EEG data was firstly down-sampled to 200 Hz. In all the following filtering steps, onepass-zerophase Hamming windowed sinc finite impulse response filters were used (Widmann et al., 2015). The downsampled data was subsequently filtered with a 0.3 Hz high-pass (transition width 0.6 Hz, order 1,100) and 45 Hz low-pass (transition width 8 Hz, order 84) filter for time-frequency analysis and 30 Hz low-pass (transition width 7.5 Hz, order 88) filter for the ERP analysis. In order to clean the data from artifacts, we performed the independent component analysis (ICA) decomposition using an extended infomax algorithm (Lee et al., 1999). The ICA was performed on a differently high-pass filtered data to get a better SNR and classification accuracy (Winkler et al., 2015) following these steps: The original down-sampled data was filtered with a 1.5 Hz high-pass (transition width 1 Hz, order 660) and 45 Hz low-pass (transition width 8 Hz, order 84) filter and segmented into 2 s epochs. Trials with excessive artifacts having peak-to-peak voltage differences exceeding 400 μV (after excluding frontal channels to keep trials with eye blinks) were removed from the data. ICA was applied and the components corresponding to eye blinks, lateral eye movements and heartbeats were identified by visual inspection. The ICA demixing matrix was then applied to the original continuous data (0.3–45 Hz filtered data for time-frequency analysis and 0.3–30 Hz filtered data for ERP analysis). Afterward, the previously identified artifactual components were removed before back-projecting the components onto the source space. On average, 5.8 ± 0.62 out of 64 independent components were excluded.

After ICA cleaning, the continuous EEG data was segmented by taking 4,600 ms pre- and 1,900 ms post responses and 2,800 ms pre- and 3,700 ms post stimulus onset and subsequently categorized into different trial types according to the identified criteria in previous sections. Hit trials were aligned to both stimulus presentation times and response execution times. Misses were aligned to stimulus presentation times. FAs and BPs were aligned to response execution times. CRs were not aligned to any external event but to the beginning of each trial. Following automatic removal of the trials containing peak-to-peak amplitudes higher than 300 μV, the trials were visually inspected and artifactual trials were excluded from further analysis. The number of artifactual trials that were removed for different trial types were as follows: hits: 2.94 ± 3.03, FAs: 1.88 ± 2.06, misses: 1.75 ± 1.92, which resulted in the following number of remaining trials for EEG analysis: hits: 107.86 ± 17.44, FAs: 66.87 ± 32.42, misses: 56.85 ± 16.20.

##### Event-Related Potential (ERP)

We performed standard ERP analysis for different types of trials. EEG responses belonging to hits and misses, were aligned with respect to the stimulus onset. In addition, in order to have a reference for investigating the FAs with no stimulus being present, the hits were also aligned to response execution times. For plotting purposes, the extracted ERPs were baseline-corrected using a baseline window of 200–0 ms prior to stimulus-onset for the stimulus-aligned ERPs and −3,100 to −2,900 ms prior to response execution for the response-aligned ERPs.

##### Time-Frequency Analysis

Wavelet analysis was performed on single trials and the absolute values of the wavelet transform were then averaged following the definition of ERSPs by Makeig (1993). For this reason complex Morlet wavelets, defined as complex sine waves tapered by a Gaussian were used. The frequencies of the wavelets ranged from 1 to 45 Hz and the number of cycles increased linearly from 3 to 10 cycles (denoted by *n* in the formulas). The temporal full-width at half-maximum (FWHM), also called as full-duration at half-maximum (FDHM = $\frac{n\,\sqrt{2\,\mathrm{ln2}}}{\pi \,f}$) ranged from 1,124 to 83 ms with increasing wavelet peak frequency. This resulted in a spectral FWHM ($\frac{2\,\sqrt{2\,\mathrm{ln2}}\times f}{n}$) ranging from 0.78 to 10.60 Hz (Cohen, 2019). The step size of the sliding window was 10 ms and the spectral resolution was interpolated to 0.2 Hz. The extent of temporal smearing caused by a wavelet is determined by the wavelet’s FDHM, hence, the activation and baseline periods were separated by a marginal boundary equal to FDHM at each frequency band (Iemi et al., 2017).

##### Statistical Analysis

Statistical analysis was performed in two steps. Firstly, the ERP and ERSPs in the activation intervals were compared against baseline intervals using a non-parametric cluster-based permutation test using the so-called “activation-vs.-baseline T-statistic” as implemented in fieldtrip (Maris and Oostenveld, 2007). In the next step, ERPs and ERSPs in pairwise trial categories were compared. Before comparing each pair of trial categories, the number of trials in the two sides of comparison was equalized by sub-selecting the trials of the side with higher number of trials. For this purpose, a cluster-based permutation test using a dependent samples t-statistic was conducted. In this stage, the following comparisons were made: hit (stim-locked) vs. miss, FA vs. CR, FA vs. BP (only for seven subjects) and hit (response-aligned) vs. FA.

In the first step of the cluster-based permutation test, the initial test statistic was computed, either by comparing each sample in the activation period against the corresponding time-averaged value in the baseline period, or by comparing the corresponding samples in the two comparison sides (two trial categories). The data structure was 2-dimensional samples (time × electrodes) of electric potentials for ERPs and 3-dimensional samples (time × frequency × electrodes) of power values for time-frequency spectra. The initial test statistics were thresholded at the 2.5-th and the 97.5-th quantiles. Subsequently, the selected samples were clustered into connected sets on the basis of spatio-temporal adjacency in the case of ERPs and temporal, spatial and spectral adjacency in the case of ERSPs. The maximum of the absolute sum of the test statistics within each cluster was taken as the observed cluster-level statistic. Afterward, the randomization distribution of the cluster-level statistic was approximated using the Monte-Carlo approach by randomly permuting the baseline and activation periods, or the two sides of comparison for 1,000 repetitions, independently for each participant, and calculating the cluster-level statistic for each random partition. Finally, the estimate of the permutation *p*-value was calculated as the proportion of random partitions that resulted in a larger cluster-level statistic than the observed cluster-level statistic. This *p*-value was then calculated for the next largest observed cluster-level statistics. The resulting clusters with cluster-level *p*-values below a critical alpha level of 0.05 (equivalent to a two tailed test) were considered to be statistically significant.

##### Spatial Correlation

Since ERP components are typically defined in stimulus-aligned trials, we tested for spatial correlation between the topographical distributions of the corresponding positive and negative components in stim- and response-aligned hits as a means to illustrate their similarities. The time intervals for selecting the positivity and negativity in the ERPs of each single subject were selected based on the temporal spread of the components in grand average ERPs. The positivity in stimulus-aligned hits were selected as the maximum value of ERP at electrode “Pz” in the time range from 400 to 1,300 ms post stimulus onset and the negativity was selected as the minimum in the time interval from 100 to 600 ms post stimulus onset. For response-aligned hits, this time range was from −250 to 250 ms for the positivity and from −900 to −100 ms for the negativity. In the first step, the two vectors representing the ERP amplitudes at each electrode for stimulus-aligned and response-aligned positivity and negativity in hits were correlated. Next, the correlation coefficient between response-aligned positivity and negativity in hits and FAs were computed. Additionally, correlation coefficients were calculated for the grand average ERPs.

## Results

### Behavioral Findings

As expected, with the employment of the explained auditory signal detection task, all participants had FAs, i.e., experienced task-elicited auditory illusions. Utilizing the previously specified criteria to categorize the trials, before removing artifactual trials based on EEG, the average number of hits was equal to 110.80 ± 21.15 (61% ± 12). The total number of misses was equal to 58.6 ± 19.22 (32% ± 10). The total number of FAs had a larger variability among participants and was equal to 67.85 ± 51.61 (Figures 2C,D). It should be noted that the sum of hits and misses in percentage do not add up to 100% since we did not include the trials which were categorized in the margin that we set to distinguish hits and misses. In addition, the rate of FAs in percentage is not reported since the total number of signal absent trials is not defined in our experiment due to the continuous design. Three subjects with fewer than 10 FAs were excluded from the EEG analysis.

### ERP

**FIGURE 3:**
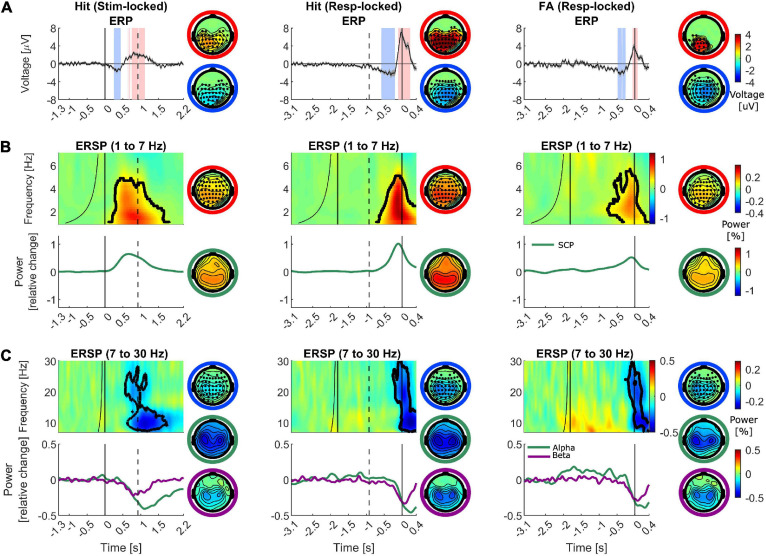
Group-averaged ERP, ERSP and spectral profiles for different frequency bands for hits and FAs. Time is denoted on the *x*-axis in seconds. The left column depicts the hits aligned to the stimulus onset, the middle column illustrates the data corresponding to hits aligned to the response execution and the right column exhibits FAs (aligned to the response time). The dashed vertical line marks the median reaction time in the left column and the median stimulus onset in the middle column. The scalp topographies with marked electrodes as filled circles represent the significant cluster distributions (negative difference: blue outer circle, positive difference: red outer circle). The rest of the scalp topography maps represent the spatial spread of the half width at half prominence of the illustrated peak or trough in the left part of the panel (also color coded with the outer circle). **(A)** Baseline corrected ERPs (left: –1.3 to –1.1 s, middle and right: –3.1 to –2.9 s). The shaded areas around the ERPs denote the standard error of the mean. The shaded intervals in light red and blue mark the significant interval with positive and negative differences with respect to baseline. **(B,C)** ERSPs for each trial group are plotted for a representative channel. Frequency (in Hz) is shown on the *y*-axis. The plotted values are in units of the power change relative to the baseline power. The baseline time range at each frequency is the interval between the minimum latency illustrated in the plot and the black curve. The time range between the curve and the straight line to its right marks the marginal boundary between the baseline and the activation intervals accounting for the temporal smearing caused by the wavelets’ temporal width. The significant cluster for the plotted electrode is marked by the black contour. **(B)** Top row: ERSPs are plotted for the frequency range of 1–7 Hz for electrode “Pz” as a representative of the time-frequency spectral pattern. Bottom row: The Spectral power traces plotted for SCP range. **(C)** Top row: average ERSPs are plotted for the frequency range of 7–30 Hz for electrodes “C3” and “C4” as a representative of the time-frequency spectral pattern. Bottom row: The Spectral power traces plotted separately for alpha and beta frequency bands.

In the first step, standard ERP analysis and the cluster-based permutation statistical test were performed to compare the electric potential differences in the activation period vs. baseline. Figure 3A illustrates the grand-averaged ERPs for hits (left and middle columns) and FAs at electrode “Pz” (right column). The light red and blue shaded areas illustrate the significant intervals with positive and negative differences. The scalp topography on the top row illustrates the spatial distribution of the significant positive cluster, as the average of the voltages in the interval that is marked as significant for each channel divided by the total number of time points and the scalp topography in the bottom row illustrates the same distribution for the significant negative cluster. The marked channels with filled circles are part of the significant cluster. As plotted, the stimulus-aligned hits’ ERP went through an initial dip following the stimulus presentation around 345 ms (−2.1 ± 1.5 μV at “PO4,” *p* = 0.004), and subsequently increased to peak around 800 ms (2.5 ± 1.4 μV at “Pz”). This peak had a centro-parietal scalp topography, resembling a P300 scalp distribution. The results of the statistical test revealed a significant channel-time positive cluster (*p* = 0.002) with a centro-parietal topography around this peak. Similarly, the response-aligned hits’ ERP underwent a slower decrement around −250 ms (−2.7 ± 2.5 μV at “CP4,” *p* = 0.002) followed by a sharper and stronger peak at −25 ms pre-response with an amplitude of 7.5 ± 3.1 μV (*p* < 0.001) happening shortly before the response execution with a centro-parietal scalp distribution. Likewise, the gradual decrease around −415 ms (−2.9 ± 1.9 μV at “CPz,” *p* < 0.001) followed by an increase to a peak, albeit weaker, with centro-parietal topography was observed in ERPs for FAs. The highest peak occurred at “POz” at −15 ms pre-response with amplitude 4.2 ± 2.6 μV (*p* = 0.003). The ERPs corresponding to misses (*N* = 16, Supplementary Figure 1A) and BPs (*N* = 7, Supplementary Figure 1B) did not reveal any significant activity changes relative to the baseline period.

**FIGURE 4:**
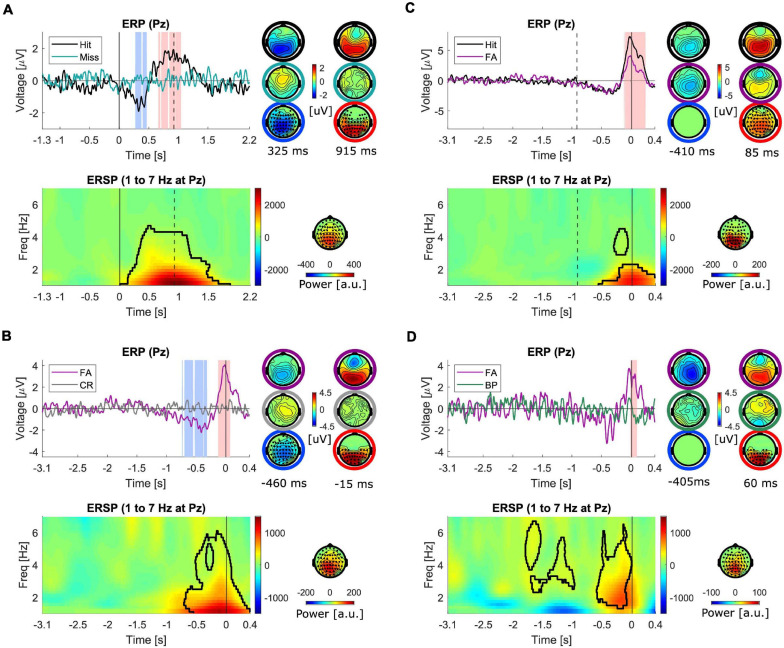
Comparison of ERPs and ERSPs (1–7 Hz) between different trial types. Time is denoted on the *x*-axis in seconds. ERSP plots reflect differences between the two conditions. All the plots represent the signals at electrode “Pz.” The shaded intervals in light red and blue mark the significant interval with positive and negative differences between the two conditions. The topographies on the right of the ERPs represent the scalp distribution of the voltages corresponding to the two conditions separately (top two rows of topographies) at the time with strongest negative (left) and strongest positive differences (right) and the differences between conditions (bottom row of topographies). The marked electrodes with filled circles are part of the significant cluster. The black contour in the ERSPs marks the significant cluster and the topography maps show the scalp distribution of the significant cluster. **(A)** Hit-miss, aligned to stimulus-onset (*N* = 16). The dashed vertical line marks the median reaction time. **(B)** FA-CR, aligned to response time (*N* = 16) **(C)** Hit-FA, aligned to response time (*N* = 16). The dashed vertical line marks the negative median reaction time **(D)** FA-BP, aligned to response time (*N* = 7).

In the next step, the ERPs in different conditions were compared. The first comparison was made between hits and misses (Figure 4A). Both of these trial types contained stimuli which resulted in responses, i.e., conscious perception, in hits but not in misses. The ERPs are plotted at electrode “Pz” in Figure 4. As illustrated, the ERP corresponding to misses is almost flat, hence both the earlier negative dip (*p* = 0.003) and the later positive peak (*p* < 0.001) in hits turn out to be significantly different than misses. The topographies on the right (right column) represent the scalp distribution of voltages at the time of the highest negative difference (*t* = 325 ms), (top: in hits, middle: in misses) and the scalp distribution of the significant negative differences (bottom). The negative differences exhibited a centro-parietal topography. The left column shows the corresponding scalp distribution of voltages at the time of the highest positive difference (*t* = 915 ms), (top: in hits, middle: in misses) and the scalp distribution of the significant positive differences (bottom).

Subsequently, FAs and CRs were compared (Figure 4B). Both of these trial types contained no stimuli, but one type (FA) prompted responses, i.e., conscious perception. As illustrated, the ERP corresponding to CRs is almost flat, hence both the earlier negative dip (<0.001) and the later positive peak (*p* = 0.003) in FAs turn out to be significantly different than CRs. The topographies on the right (right column) represent the scalp distribution of voltages at the time of the highest negative difference (*t* = −460 ms), (top: in FAs, middle: in CRs) and the scalp distribution of the significant negative differences (bottom). The left column shows the corresponding scalp distribution of voltages at the time of the highest positive difference (*t* = −15 ms), and the scalp distribution of the cluster of significant positive differences (bottom). The topographies of both positive and negative differences exhibited similar scalp distributions to differences of hits vs. misses. In addition, in order to control for the effect of manual pressing of the response button, FAs were compared against BPs for a subset of subjects (*N* = 7, Figure 4D). The positive difference with a maximum at 60 ms remained significant (*p* = 0.019) but the negative difference did not reach the significance level (*p* = 0.099).

Finally, the activity in hits and FAs were compared. The results of previous comparisons showed that both hits and FAs exhibited an early negativity and a late positivity when compared against misses and CRs, respectively. In order to check whether the magnitudes of these two components differed in hits and FAs, i.e., to examine the differences between veridical and illusory perceptions, they were directly compared against each other (Figure 4C). The results of this comparison showed that the positivity in hits was significantly higher than FAs (*p* < 0.001) while the negativity appeared not to be significantly different. In fact, the negativity appeared to have a similar amplitude, latency and scalp distribution in both conditions.

**FIGURE 5:**
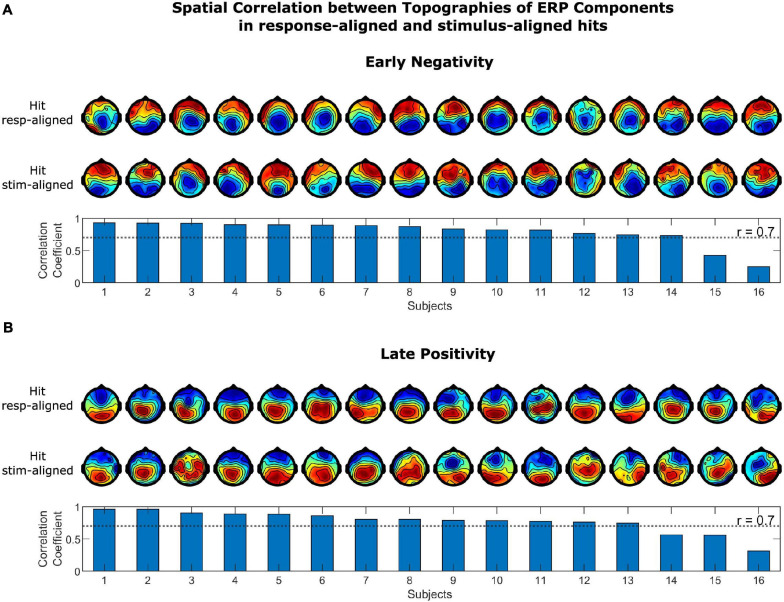
Spatial correlation between topographies of ERP Components in stimulus-aligned and response-aligned hits for **(A)** the early negativity and **(B)** the late positivity. Topographies in the upper row correspond to response-aligned hits and in the lower row to stimulus-aligned hits. Scalp topographies of the components were highly correlated in stimulus-aligned and response-aligned hits across most of the subjects.

**FIGURE 6:**
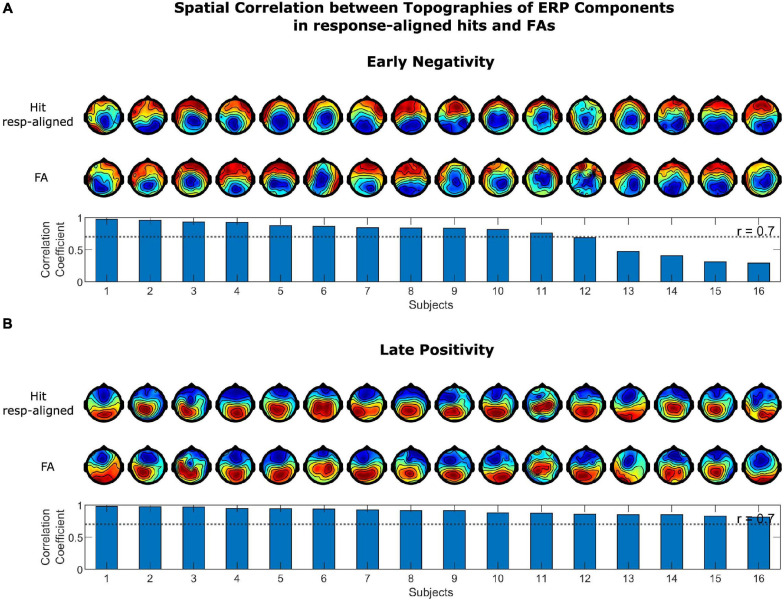
Spatial correlation between topographies of ERP Components in response-aligned hits and FAs for **(A)** the early negativity and **(B)** the late positivity. Topographies in the upper row correspond to response-aligned hits and in the lower row to FAs. Scalp topographies of the components were highly correlated in response-aligned hits and FAs across most of the subjects.

The spatial correlation of the topographies of the LP and EN in stimulus-aligned and response-aligned hits is illustrated in Figure 5. The correlation coefficients for the EN ranged between 0.250 and 0.929, which for 14 out of 16 subjects were above 0.7. The correlation coefficient for the LP ranged between 0.316 and 0.965, which were above 0.7 for 13 out of 16 subjects. The correlation coefficients of LP and EN for the grand average ERPs were 0.970 and 0.866, respectively. The spatial correlation of the topographies of the LP and EN in response-aligned hits and FAs is illustrated in Figure 6. The correlation coefficients for the EN ranged between 0.291 and 0.974, which for 11 out of 16 subjects were above 0.7. The correlation coefficient for the LP ranged between 0.811 and 0.980, which were above 0.7 for all of the subjects. The correlation coefficients of LP and EN for the grand average ERPs were 0.964 and 0.887, respectively.

### ERSP

Given that previous studies have pointed toward the role of the low-frequency activities such as SCP in generating conscious perception (Karakaş et al., 2000; Andrew and Fein, 2010; Bachman and Bernat, 2018; Popp et al., 2019) and the role of ∼3–4 Hz cortical power in encoding auditory object boundaries (Riecke et al., 2009, 2012; Kaiser et al., 2018), we investigated the time-frequency representation of EEG epochs in different trials in the lower frequency range. First, the ERSPs in the 1–7 Hz frequency range were calculated and relevant statistical tests were performed to compare the spectral power values in the activation vs. baseline intervals. In Figure 3B, upper row, ERSPs for the illustrative electrode “Pz” for hits and FAs are plotted and the black contour in the time-frequency plots marks the significant cluster distribution. The scalp topography on the right of the time-frequency plots illustrates the spatial distribution of the significant positive cluster as the average of the spectral power values in the time-frequency region that is marked as significant for each channel. The result of wavelet analysis revealed a power enhancement concentrated in the 1–5 Hz frequency range in both hits and FAs which also turned out as statistically significant compared to baseline. The positive cluster in the stimulus-aligned hits, response-aligned hits and FAs had a *p*-value of 0.004, 0.006, and <0.001, respectively. The average activity in the 1–4 Hz frequency range corresponding to SCP, is plotted in the bottom row. This activity exhibited a gradually increasing trajectory which peaked shortly before the median reaction time in stimulus-aligned hits and before the response time in response-aligned hits as well as FAs with a parietal topography that extends to frontal regions.

Pre-stimulus alpha and to a lesser extent beta frequency bands have been implicated in the formation of illusions in previous studies, therefore, we also examined the ERSP for alpha and beta frequency bands (Lange et al., 2014; Leske et al., 2014; Schepers et al., 2016). The ERSPs in the 7–30 Hz frequency range were calculated and the spectral power values in the activation vs. baseline intervals were statistically compared. In Figure 3C, upper row, average ERSPs for the average of electrodes “C3” and “C4” for hits and FAs are plotted and the black contour in the time-frequency plots marks the significant cluster distribution. As shown, a significant alpha to beta event-related desynchronization (ERD) is identifiable across a large number of sensors near the sensorimotor cortices. The significant cluster extended through both alpha and beta ranges (*p* < 0.001 in stimulus-aligned hits, *p* = 0.007 in response-aligned hits, *p* < 0.001 in FAs). In the separate alpha (8–12 Hz) and beta (15–25 Hz) power trajectories, both band powers underwent a gradual decrease in line with neural signatures of motor response planning. The alpha suppression started around 370 ms and reached its lowest value of −0.42 uV around 1,100 ms post-stimulus onset in stimulus-aligned hits. For response-aligned hits, the suppression started around −540 ms pre-response and reached the lowest value of −0.46 uV at 270 ms post-response at “C3.” For FAs, the suppression started around –700 ms pre-response and reached the lowest value of –0.4 uV at 80 ms post-response at “C3.” The beta suppression exhibited a faster dynamic than alpha suppression. It started around 370 ms post-stimulus onset and reached its lowest value of −0.24 uV around 800 ms post-stimulus onset in stimulus-aligned hits at “CP3.” For response-aligned hits, the suppression started around −600 ms pre-response and reached the lowest value of −0.34 uV at −110 ms post-response at “CP3.” For FAs, the suppression started around −240 ms pre-response and reached the lowest value of −0.32 uV at 70 ms post-response at “CP4.” Both alpha and beta ERDs exhibited bilateral topographies with contralateral dominance (not the case in beta activity for FAs). The ERSPs corresponding to misses (*N* = 16, Supplementary Figure 1A) and BPs (*N* = 7, Supplementary Figure 1B) did not reveal any significant activity changes relative to the baseline period.

In the next step, as for ERPs, the ERSPs in different conditions were compared. A significant power enhancement in the frequencies below 4 Hz in hits compared to misses (*p* = 0.008, Figure 4A) as well as in FAs compared to CRs (*p* = 0.012, Figure 4B) were found. The power enhancement in FAs was also significantly different than in BPs (*p* = 0.002, Figure 4D) for the available subset of subjects with this control condition. The scalp distribution of the significant clusters was similar and mainly distributed over parietal regions slightly extended to fronto-central areas. Finally, the comparison of hits vs. FAs revealed a significant power enhancement mainly in frequencies below 2 Hz (*p* = 0.044, Figure 4C), with a parietal scalp distribution. The results of the comparison of the ERSPs in pairwise trial categories for the higher frequency range are presented in the Supplementary Material, since they did not reveal any activations corresponding to FAs different than motor-related activity as captured in BPs.

## Discussion

In previous studies, different experimental paradigms have been utilized to induce different forms of auditory illusions, however, the number of studies investigating the neural correlates of speech in noise auditory illusions are relatively sparse (Schepers et al., 2016). The goal of our experiment was to induce task-elicited auditory illusions in our group of participants in order to study their corresponding neural underpinnings. To this end, we studied EEG responses in FAs, which we believe represents task induced auditory illusions in a speech in noise signal detection task. Our results suggest that illusory percepts as reflected in FAs show ERP and oscillatory responses similar to real percepts albeit with sometimes reduced amplitudes.

### Behavioral Findings

The behavioral results show that all of the subjects taking part in the experiment, without a history of psychotic disorders, had FAs, i.e., task-elicited auditory illusions. This observation has been reported in previous studies with similar tasks, which either directly experimented non-clinical samples (Pries et al., 2017) or studied their behavior in the control group (Vercammen et al., 2008; Galdos et al., 2011). The variability in the number of FAs, or the response bias across subjects have been attributed in previous studies to individual differences in factors such as trait suggestibility (Inventory of Suggestibility, IoS), hallucination proneness (Launay–Slade Hallucination Scale; LSHS) (Alganami et al., 2017), or to positive schizotypy (Schizotypal Personality Questionnaire; SPQ) (Galdos et al., 2011; Gonzalez de Artaza et al., 2018). Nevertheless, these relationships are not yet well-established and further research is needed to elucidate the relationship between different traits such as hallucination proneness in non-clinical individuals and signal detection theory (SDT) parameters (Pries et al., 2017).

### Experimentally-Induced Auditory Illusions

Signal and voice detection tasks, similar to our design, have been found to be the most robust paradigms to experimentally-induce auditory perceptual experiences (Anderson et al., 2020) and have been employed in various studies for this purpose (Bentall and Slade, 1985; Barkus et al., 2007, 2011; Vercammen et al., 2008; Moseley et al., 2014). Additionally, an imbalance between perceptual expectation and external input, has been proposed to mediate hallucinatory experiences (Chhabra et al., 2016). This manipulation can be exercised by means of various experimental procedures such as varying the level of semantic expectation of the auditory signal (Vercammen and Aleman, 2010; Hoskin et al., 2014) or Pavlovian conditioning (Powers et al., 2017).

In the current experiment, we introduced an ambiguity in the auditory stream by presenting the speech snippets at each individual’s detection threshold intensity and masking them by noise with similar spectral profile. This bottom-up processing ambiguity was accompanied by using a manipulation of expectation of subjects to hear a speech snippet through changing the frequency of presentation of the speech stimuli. To be more specific, alternating periods of shorter and longer ISIs, increased the expectancy of the subjects to hear speech snippets in the periods with longer ISIs, thus increasing the experience of auditory illusions possibly through top-down control mechanisms. Since Hallucinations and illusions have been postulated to be generated by top-down effects on perception which are mediated by inappropriate perceptual priors (Aleman et al., 2003; Vercammen and Aleman, 2010; Chhabra et al., 2016; Powers et al., 2016, 2017; de Boer et al., 2019), we believe that increasing expectation in our experiment by manipulating ISIs, largely prompted auditory illusions and to a lesser extent led to purely decision-based responses. This can be further supported by the findings that activations in the auditory cortex were found similarly during hallucinations and FAs (Barkus et al., 2007) and that individuals with experience of auditory hallucinations, had higher FAs in auditory signal detection/recognition tasks compared with individual without such experiences (Bentall and Slade, 1985; Barkus et al., 2007, 2011; Chhabra et al., 2016; de Boer et al., 2019).

As the purpose of the present study was to investigate the neural processes associated with false auditory perceptions, having a sufficient number of FAs to achieve a sufficiently high EEG signal to noise ratio was necessary. Previous research affirms that a continuous design does not limit the maximum possible number of FAs and is less prone to ceiling effects (Huque et al., 2017). In addition, this design enables investigating of the neural activity preceding the FAs over longer periods of time. Therefore, instead of a discrete trial design, with predefined trial and response intervals, we employed a continuous design.

However, a continuous design has some disadvantages with regard to the analysis of the acquired data. The first difficulty arises in categorizing the trials into different types. Due to the lack of discrete trials with a predefined response interval, the valid time interval for responding to the stimuli needed to be chosen *post-hoc* and empirically based on the distribution of the response times to the speech snippets. A too liberal criterion to categorize the trials as hits leads to mistakenly attributing late responses to previously presented stimuli, thus incorrectly marking a FA as a hit. On the other hand, a too conservative criterion leads to assignment of the late response to a FA, whereas it was in fact triggered by the preceding stimuli, and at the same time incorrect categorization of the previous stimulus-present trial as a miss instead of a hit (Huque et al., 2017). In order to reduce the likelihood of categorizing the trial types erroneously, a marginal boundary was chosen for the criteria for classifying hits and FAs. This strategy was at the expense of losing the trials that were lying in the marginal boundary range but gaining a higher confidence in trial classification. Another disadvantage arising due to the lack of a predefined interval to collect the responses of the subjects, is that the time windows of perception and action preparation in hits and FAs overlaps. While this is beneficial to follow how perception transforms into action, it renders distinguishing the two processes challenging. Comparing CRs, as the trials without a speech snippet and no response, with FAs would only determine the processes underlying both perception and action processes together. For this reason, we also compared FAs against BPs to control for movement-related effects for a subset of subjects.

### Early Negativity and Late Positivity Components in ERP

As illustrated, a negativity in ERPs of stimulus-aligned hits was observed which was absent when these trials were compared against misses and shared a high topographical correlation to the negativity in response-aligned hits across all subjects. A negativity having a similar temporal and spatial distribution was also observed in FAs. Previous studies demonstrated the presence of a negativity in aware minus unaware trials in a threshold-intensity tone awareness task, which was called AAN (Eklund and Wiens, 2019) and resembled visual awareness negativity (VAN) in vision (Koivisto and Revonsuo, 2010). This negativity has been suggested to be an early neural correlate of awareness in vision and hearing. Due to the lack of individual anatomical scans of the subjects, we did not perform source localization but the results of source localization in previous studies suggested that AAN originates from bilateral auditory cortices (Eklund et al., 2019, 2020). Thus, AAN has been postulated to be the neural correlate of localized recurrent processing that occurs within early areas of the sensory cortices. Recurrent processing results from horizontal connections as well as feedback from higher cortical areas and has been suggested to be necessary, on top of feedforward information transfer to generate awareness (Lamme and Roelfsema, 2000; Eklund et al., 2019). If the negativity in the present study in stimulus-aligned hits, and the similar negativities in response-aligned hits and FAs, represent the same or a similar component as AAN, which can be elucidated further in future studies with source localization, the current findings demonstrate that local recurrent processing in the auditory cortex occurred in both hits and FAs, i.e., veridical and illusory perceptions. Local recurrent processing in the auditory cortex in the absence of an external stimulus might have been caused by top-down feedback from higher cortical areas, exerted due to higher expectations to hear an auditory stimulus, which amplified the ongoing activities in the auditory cortex related to processing of the masking noise.

The next observable component in the ERP trajectories in our experiment is a later positive peak in centro-parietal regions peaking around 800 ms post-stimulus onset in the stimulus-aligned hits and shortly before the button-press in response-aligned hits and FAs. The spatio-temporal characteristics of this component is in line with P300 specifications. The classic P300 component is characterized as a large positive deflection in the ERP with a centro-parietal scalp topography that occurs approximately during the 250–500 ms time interval after the stimulus onset (Polich, 2007). The more recent accounts of P300 propose an alternative explanation of it as an evolving signal reflecting the dynamics of the decision process rather than a unitary event (Kelly and O’Connell, 2013, 2015; Twomey et al., 2015). Often referred to as the CPP, this signal accumulates sensory evidence up to the decision boundary threshold, the moment at which the stimulus is consciously perceived and the decision is made (Kelly and O’Connell, 2015). This signal returns to baseline after making the decision, hence a peak is formed. CPP has been suggested to be equated with P300 (O’Connell et al., 2012).

The larger amplitude of the CPP in response-aligned hits as compared to stimulus-aligned hits is in accordance with previous findings (O’Connell et al., 2012). This can be explained by considering that as the postulated signal encoding the decision variable, CPP/P300 should be more closely tied to the response than to the stimulus onset (Kelly and O’Connell, 2015). The smoother and lower amplitude peak in stimulus-aligned hits can be explained as being the result of high variability of reaction times which imposes differently timed trajectories for CPP (Kelly and O’Connell, 2015). This variability is largely diminished in response-aligned trials, thus exhibiting a narrower width and higher peak amplitude. This neural pattern was absent in misses where the presented stimuli were not consciously perceived by the subjects (*N* = 16, Supplementary Figure 1A) and in BPs where motor responses were given without the presence of any underlying perceptual experience (*N* = 7, Supplementary Figure 1B).

A similar positivity is identifiable in FAs. In agreement with our findings, previous studies also reported the presence of a reliable build-up in CPP in FAs, suggesting it to be the result of an excessive accumulation of internal sensory noise (O’Connell et al., 2012). Consistently, it has also been reported that CPP closely tracks the subjective perceptual evidence, over and above the physically presented evidence in a visual discrimination task (Tagliabue et al., 2019).

As illustrated in Figure 4C, this positivity reached a lower peak amplitude in FAs compared to hits, similar to previous findings (O’Connell et al., 2012). If the peak amplitude represents the decision threshold, this observation can be explained by induced shifts in the decision criterion as a result of alterations in the prior expectation of occurrence of the speech snippets. An alternative explanation might be that CPP is reflecting the confidence rather than the decision variable. The decision variable is the signal on which the decision rule is applied and the decision is made (Shadlen and Kiani, 2013). Therefore, its value at the time of response should not be affected by the confidence of the subjects in making the decision. On the other hand, confidence reflects the certainty of the subject in responding, and could be reflected in the distance between the decision variable and the decision bound (Urai and Pfeffer, 2014; Kelly and O’Connell, 2015). If the CPP encodes confidence, it should be lower when the sensory evidence is weaker, i.e., the more difficult hit trials should exhibit a lower amplitude of CPP. Additionally, since subjects are less certain about their decisions in FAs due to the absence of sensory stimuli and noisier sensory sampling compared to hits, the signal encoding confidence should be lower in FAs compared to hits at the moment when the decision is made (Urai and Pfeffer, 2014). The lower amplitude of ERPs in FAs compared to hits in our results provide evidence supporting that CPP encodes confidence rather than the decision variable. This is in line with the findings of a vibrotactile frequency comparison study, which showed that the peak of CPP did not reach fixed threshold, and therefore seemed to reflect decision confidence (Herding et al., 2019).

Other short-lived ERP components require a discrete, sudden-onset and high intensity stimuli to become identifiable. Otherwise, their amplitude cannot be distinguished from uncorrected noise in EEG signals or they will be suppressed or canceled out due to the overlap of the positive and negative peaks in different trials. The speech snippets in our experiment were Hann smoothed at the onset and offset and were presented at near detection threshold intensity. Hence, the highest amplitude peak in each speech stimulus, might have been the only perceived part of the stimulus, which did not have a fixed latency across different stimuli, and occurred variably within the 1-s duration of each stimulus, in addition to having a low intensity. This explains the absence of the transient auditory evoked signals in the standard ERP.

### ERSP

Our results indicate a power enhancement in frequencies up to ∼5 Hz in both hits and FAs, which was absent in CRs, BPs, and misses (Figure 4 and Supplementary Figure 1). The widely distributed activity below 4 Hz, being referred to slow cortical potentials (SCPs), has been postulated to be a correlate of conscious awareness (He and Raichle, 2009). SCP and P300 have been suggested to be related to each other in such a way that P300 is part of the SCP family (Li et al., 2014). It has also been repeatedly demonstrated that P300 is associated with activities in delta and theta frequency bands (Başar-Eroglu et al., 1992; Demiralp et al., 2001; Schürmann et al., 2001; Güntekin and Başar, 2016). The findings of the present study suggest that low-frequency activity, as suggested to be a correlate of conscious perception, is also present in false perceptions in the absence of external stimuli.

An additional function associated with the observed power enhancement especially in the ∼3–4 Hz range can be related to the detection of auditory edges which is an essential step in auditory signal processing (Fishbach et al., 2001). Failure to detect salient edges in the auditory stream was found to be accompanied by suppression in the ∼3–4 Hz range (Riecke et al., 2009; Kaiser et al., 2018). These findings can be regarded as an alternative or additional function associated with the power enhancements in the ∼3–4 Hz range. However, further studies accompanied by source localization are needed to investigate this hypothesis more accurately.

Alpha power modulations in association with certain forms of task-elicited illusions in different sensory modalities have been observed in previous studies (Lange et al., 2014; Leske et al., 2014; Schepers et al., 2016). Additionally, studies mainly in the visual domain demonstrated that reduced prestimulus alpha power increases the likelihood of perceiving an illusory visual stimulus, however, it has been proposed that alpha has a more general effect on perceptual decisions across sensory modalities by modulating the neural excitability and therefore the sensory bias (rather than sensitivity) (Samaha et al., 2020). Our results did not provide evidence for the involvement of alpha power modulations in the generation of auditory illusions. Although ERSPs in the alpha band exhibited a significant alpha power suppression during FAs (Figure 3C), when comparing FAs against BPs, no significant difference was observed between these two conditions (Supplementary Figure 2). Other methods of analysis as in Iemi et al. (2017) might be necessary to reveal other relationships between alpha power and SDT measures such as criterion. However, our experiment was not suited for this type of analysis due to its continuous design, unknown number of signal-absent trials, as well as relatively low number of trials that is necessary to achieve reliable results for the binning analysis (Iemi et al., 2017). In general, we think that discrete trial designs, with a separate interval for acquiring responses are more suitable to capture prestimulus alpha oscillations, since due to the anatomical localization of the auditory cortex in the supratemporal plane, detecting alpha power modulations due to direct auditory processing compared to easily identifiable sensorimotor alpha ERD in EEG recordings is very challenging in case of temporal overlap (Pfurtscheller and Lopes da Silva, 1999).

Beta power has been shown to be implicated in generating illusions in a few number of studies (Keil et al., 2014; Leske et al., 2014). Similar to the alpha band, in the beta band, a significant power suppression was found starting a few 100 ms before the decision report in FAs (and hits), however, the comparison of FAs vs. BPs did not reveal any significant differences between these two conditions in this frequency band. These results suggest that the beta suppression in FAs, is mainly related to motor related beta-band ERD which can be observed during hand and finger movements (Pfurtscheller and Lopes da Silva, 1999; Hari, 2006; Cuellar and Del Toro, 2017).

### Limitations and Future Directions

The main limitation of our study is that given the continuous design, discerning between motor preparation and auditory processing is challenging since the participants were instructed to freely press the button anytime they heard a speech stimulus. Employing a discrete design with predefined intervals for responding with a substantial temporal distance can be an alternative option for future experiments but it should be noted that this requires a considerably longer task duration in order to achieve a sufficient number of FAs for a satisfactory SNR. To correct for this limitation in the current study, a subset of participants performed random button-presses to control for motor-related activities. The small sample size of the control condition in our experiment limited the reliability of some of our results. On one hand, the results of the comparison of activation vs. baseline activities in the BP condition, did not exhibit any early negativity or late positivity in ERPs or any low-frequency power enhancements in ERSPs as in FAs and only revealed a power suppression in the alpha and beta frequency ranges (Supplementary Figure 1B). However, the test for FAs was performed for 16 subjects, while for BPs only 7 subjects were available, which might have potentially limited the power of the statistical test to identify the effects in BPs. On the other hand, the early negativity in FAs was not revealed as significantly different than in BPs when these two conditions were compared (Figure 4D). This might as well be associated with the small sample size available for this test. Extending the sample size in future studies has the potential to provide further evidence for the absence or presence of the suggested effects in the current study. Nevertheless, it should be noted that by visual inspection, even a tendency for such an effect could not be observed.

The other limitation concerns the temporal accuracy of the instances at which the sensory evidence was collected, accumulated and led to perception in both hits and FAs. As already explained, in hit trials we are not able to identify which fragment of the 1 s stimulus was perceived by the subject and in FAs there is no external stimulus present for this purpose. Therefore, identifying the sensory components of the neural activities is challenging. Additionally, since FAs are aligned to the time of button-press, our analysis is inclined to reveal neural activities that are primarily timely coupled to the response. However, this design allowed for task-induced illusory experiences purely due to expectation error in absence of any confounding stimulus, which was the goal of our experiment.

Throughout the study, we assumed that detecting a stimulus, either veridical or illusory, fully reflects the presence of subjective awareness. Detecting the presence of an externally presented stimulus has been an index of conscious perception in many studies (for a review see Dykstra et al., 2017). We believe that similarly, reporting the presence of an absent stimulus indicates the presence of conscious perception in most cases. We found that neural activations in FAs were similar to hits, although sometimes with weaker amplitudes and they were similar to previously reported neural correlates of conscious perception of auditory stimuli. However, we acknowledge that there is not a perfect equality between detection and perceptual awareness and detection only provides a good approximation for the subjective awareness (Eklund and Wiens, 2019). It has been argued in previous studies that awareness is better measured when there are more than two levels to choose from Hillyard et al. (1971). Using confidence ratings with different levels is one way to measure awareness indirectly (Li et al., 2014). The most accurate way to measure awareness is to use direct clarity ratings with several alternatives to allow subjects to rate their level of awareness (Aru et al., 2018; Eklund and Wiens, 2019; Tulver et al., 2019) which can be incorporated in future experiments.

Finally, the association between the psychological traits, that have been postulated to be correlated with the susceptibility to make FAs in an auditory signal detection task, and the number of FAs was not investigated in the current study. Future studies that aim to elucidate the behavioral correlates of FAs in an auditory signal detection task, can assess factors such as trait suggestibility, hallucination proneness or positive schizotypy by means of suitable questionnaires and correlate them with the number of FAs or the response bias in similarly designed experiments. Establishment of such relationships has the potential to provide additional measures for the assessment of these factors in addition to the commonly used questionnaires in future.

## Conclusion

With the employed task, all the participants reported instances of hearing speech snippets in the absence of externally presented speech stimuli. The results of EEG analysis demonstrated that an early negativity similar to AAN and a late positivity similar to P300, in addition to a low-frequency power enhancement, all previously involved in perceptual awareness, were present in similar manners in hits, i.e., veridical as well as FAs, i.e., task-elicited illusory perceptions. Our results did not provide any evidence for the involvement of pre-stimulus alpha and beta frequency band powers in the generation of FAs. Further research is needed to investigate which neural mechanisms play a causal role in the initiation and final generation of the task-elicited auditory illusory perceptions and to further establish the link to analogous experiences in pathological conditions.

## Data Availability Statement

The raw data supporting the conclusions of this article will be made available by the authors, without undue reservation. Additionally, the experiment scripts can be accessed in the OSF repository from the following link: https://osf.io/kb9c8.

## Ethics Statement

The studies involving human participants were reviewed and approved by the Commission for Research Impact Assessment and Ethics, the University of Oldenburg. The participants provided their written informed consent to participate in this study.

## Author Contributions

MF: conceptualization, methodology, data acquisition, analysis, visualization, writing, and original draft. FK: analysis, writing, review, and editing. GA: data acquisition and analysis. AA, BĆ-B, and CH: conceptualization, methodology, writing, review and editing, funding acquisition, and supervision. All authors contributed to the article and approved the submitted version.

## Conflict of Interest

CH received honoraria as an editor from Elsevier Publishers, Amsterdam. The remaining authors declare that the research was conducted in the absence of any commercial or financial relationships that could be construed as a potential conflict of interest.
