# Supplementary material for: Similar EEG Activity Patterns During Experimentally-Induced Auditory Illusions and Veridical Perceptions
Source: Front Neurosci. 2021 Apr 1;15:602437. doi: 10.3389/fnins.2021.602437 (PMC8047478; doi:10.3389/fnins.2021.602437)
Supplement: Supplementary file 1 [file Data_Sheet_1.docx]

Supplementary Material

# Neural activity in misses and BPs

In miss trials, the stimuli were not consciously perceived by the subjects. The results of ERP and ERSP investigation of misses did not reveal any significant activity changes in the activation period compared to baseline (Supplementary Figure 1A). In particular, the early negativity and the late positivity as identified in hits were absent in misses. Hence, although both types of trials include an externally presented stimuli, their corresponding neural activity differs. This difference can be attributed to the presence or absence of perceptual awareness as well as differences in reporting, i.e., button-presses.

In the next step, the ERPs and ERSPs for BPs in the activation versus baseline periods were compared for seven subjects. No significant differences with respect to baseline was revealed by performing this test for ERPs. For ERSPs, a significant power suppression in the alpha to beta frequency ranges was revealed by this test (p < 0.001). These results suggest that the neural activity that was found in FAs (early negativity and late positivity in ERPs, and the low-frequency power enhancement in ERSPs) were not a result of the action of pressing the button but were associated with the perceptual experience of hearing an illusory voice.

| 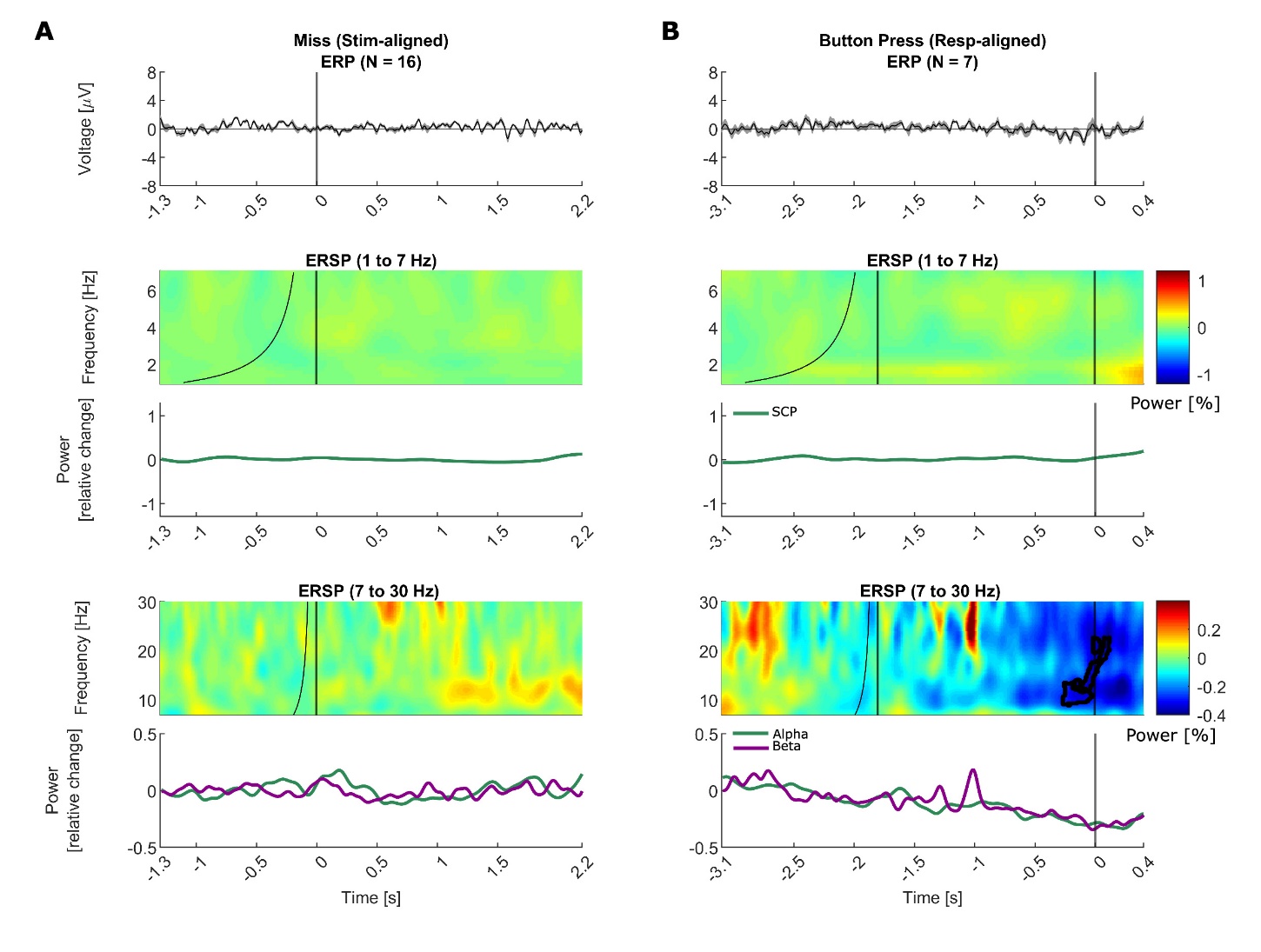 |
| --- |
| **Supplementary Figure 1.** Baseline corrected ERPs (1.3 to -1.1 s) and baseline corrected ERSPs (The baseline time range at each frequency is the interval between the minimum latency illustrated in the plot and the white curve) for (A) misses and (B) BPs. ERPs and ERSPs for 1 to 7 Hz are plotted at channel ‘Pz’ and ERSPs for 7 to 30 Hz are plotted for the average of ‘C3’ and ‘C4’. (A) No significantly different activity change with respect to baseline was identifiable for misses. (B) A significant power suppression in the alpha to beta frequency ranges in ERSPs were observed which is marked by the black contour. |

# Pairwise comparison of 7-to-30 Hz ERSPs in different trial categories

The results of the comparison of the ERSPs in paired trial categories for the 7-to30 Hz frequency range have been plotted in supplementary figure 2. A significant power suppression in the frequencies corresponding to alpha and beta bands in hits compared to misses (p = 0.002) as well as in FAs compared to CRs (p = 0.002) were found. No statistically significant differences were found between hits and FAs, as well as between FAs and BPs. Significant differences were found wherever the two sides of comparison differed in the presence or absence of reporting a perceptual experience (hit-miss and FA-CR) and no differences were identified in cases where both conditions involved button-presses (Hit-FA and FA-BP). The results of our analyses did not provide any specific differences in alpha or beta frequency band power between hits and FAs, i.e., between veridical and illusory perceptions. Additionally, no differences in the frequency range of 7 to 30 Hz were found between FAs and BPs. Therefore, the present findings did not provide any evidences for the involvement of alpha and beta power in generating FAs, i.e., auditory illusions.

| 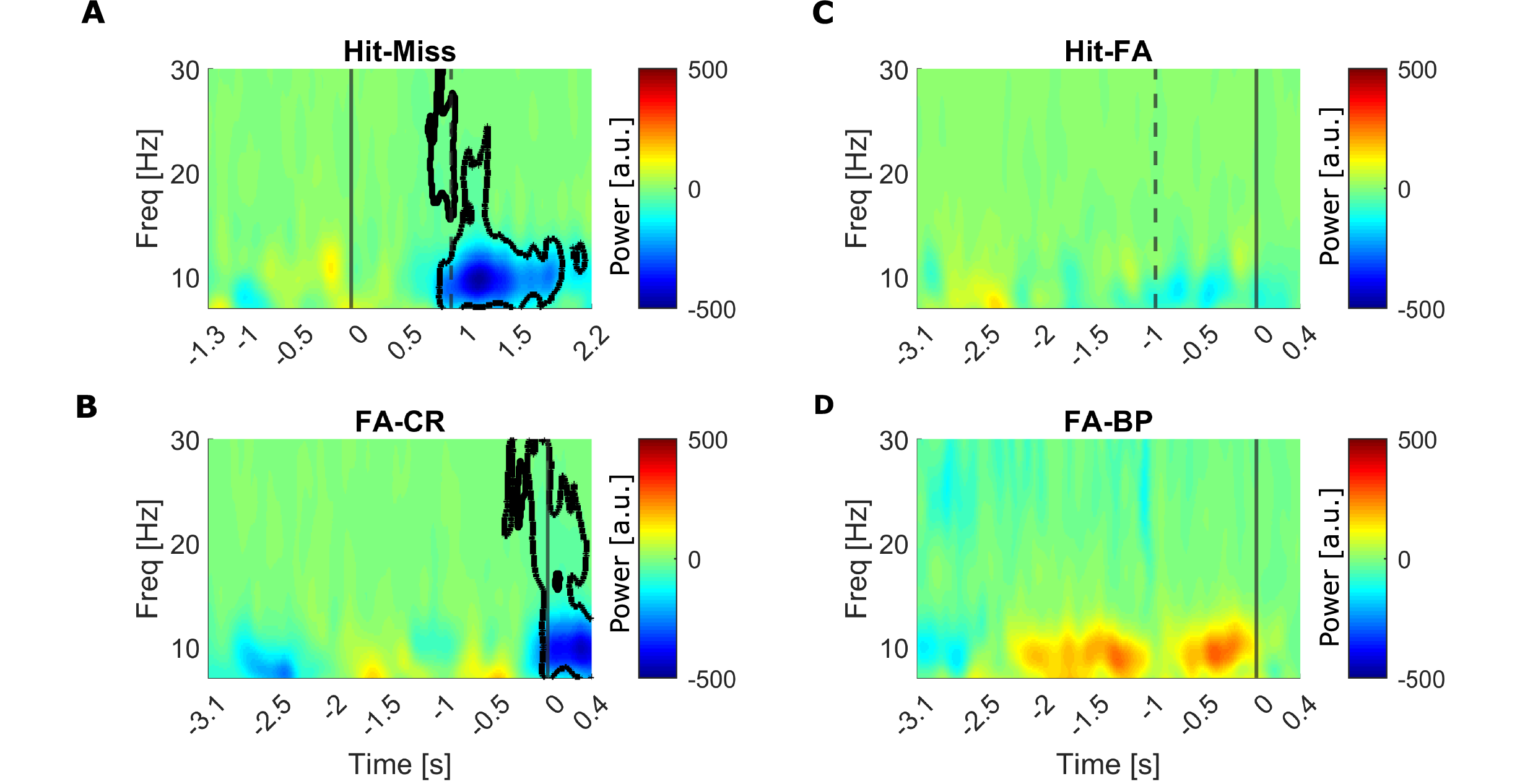 |
| --- |
| **Supplementary Figure 2.** Pairwise comparison of 7-to-30 Hz ERSPs in different trial categories. Time is denoted on the x-axis in seconds. Frequency (in Hz) is shown on the y-axis. All the plots represent the average signals of electrodes ‘C3’ and ‘C4’. The black contours in the ERSPs mark the significant cluster. (A) Hit-miss, aligned to stimulus-onset (N=16). The dashed vertical line marks the median reaction time. (B) FA-CR, aligned to response time (N=16). (C) Hit-FA, aligned to response time (N=16). The dashed vertical line marks the negative median reaction time (D) FA-BP, aligned to response time, (N=7). |
